# Efficient and effective pruning strategies for health data de-identification

**DOI:** 10.1186/s12911-016-0287-2

**Published:** 2016-04-30

**Authors:** Fabian Prasser, Florian Kohlmayer, Klaus A. Kuhn

**Affiliations:** Chair of Biomedical Informatics, Department of Medicine, Technical University of Munich (TUM), Munich, 81675 Germany

**Keywords:** Security, Privacy, De-identification, Statistical disclosure control, *k*-Anonymity, Optimization

## Abstract

**Background:**

Privacy must be protected when sensitive biomedical data is shared, e.g. for research purposes. Data de-identification is an important safeguard, where datasets are transformed to meet two conflicting objectives: minimizing re-identification risks while maximizing data quality. Typically, de-identification methods search a solution space of possible data transformations to find a good solution to a given de-identification problem. In this process, parts of the search space must be excluded to maintain scalability.

**Objectives:**

The set of transformations which are solution candidates is typically narrowed down by storing the results obtained during the search process and then using them to predict properties of the output of other transformations in terms of privacy (first objective) and data quality (second objective). However, due to the exponential growth of the size of the search space, previous implementations of this method are not well-suited when datasets contain many attributes which need to be protected. As this is often the case with biomedical research data, e.g. as a result of longitudinal collection, we have developed a novel method.

**Methods:**

Our approach combines the mathematical concept of antichains with a data structure inspired by prefix trees to represent properties of a large number of data transformations while requiring only a minimal amount of information to be stored. To analyze the improvements which can be achieved by adopting our method, we have integrated it into an existing algorithm and we have also implemented a simple best-first branch and bound search (BFS) algorithm as a first step towards methods which fully exploit our approach. We have evaluated these implementations with several real-world datasets and the *k*-anonymity privacy model.

**Results:**

When integrated into existing de-identification algorithms for low-dimensional data, our approach reduced memory requirements by up to one order of magnitude and execution times by up to 25 %. This allowed us to increase the size of solution spaces which could be processed by almost a factor of 10. When using the simple BFS method, we were able to further increase the size of the solution space by a factor of three. When used as a heuristic strategy for high-dimensional data, the BFS approach outperformed a state-of-the-art algorithm by up to 12 % in terms of the quality of output data.

**Conclusions:**

This work shows that implementing methods of data de-identification for real-world applications is a challenging task. Our approach solves a problem often faced by data custodians: a lack of scalability of de-identification software when used with datasets having realistic schemas and volumes. The method described in this article has been implemented into ARX, an open source de-identification software for biomedical data.

## Introduction

Privacy must be protected when sensitive biomedical data is shared, e.g. for research purposes. This requires implementing several safeguards [1]. Important organizational and legal measures include data use agreements and data access committees. Moreover, risks of data aggregation and sharing should already be covered in the informed consent. On a technical level, multiple layers of access can be used to create controlled environments in which it becomes possible to reason about privacy risks. These risks can then be mitigated with methods of data de-identification, which means to transform a dataset in such a way that it becomes extremely difficult for an attacker to link its records to identified individuals.

Several methods of de-identification are covered by national and international laws and regulations, such as the *US Health Insurance Portability and Accountability Act* (HIPAA) [2], and the *European Directive on Data Protection* [3]. The HIPAA *Privacy Rule* defines two basic methods [4]. The first approach requires the removal or the modification of a predefined set of 18 types of attributes. The second approach, which is called *"expert determination"* requires that a professional *"determines that the risk is very small that the information could be used [ …] to identify an individual"* [4]. This can be achieved with methods of *statistical disclosure control*, where privacy risks are measured with mathematical or statistical models [5]. As data transformation inevitably leads to loss of information and thus a decrease in data quality, a balance has to be sought between privacy risks on one side and suitability for a specific use case on the other. This means that data de-identification is a non-trivial optimization problem with two conflicting objectives.

## Background

### Transformation models

Different transformation models can be used to de-identify data. In the remainder of this article, we will focus on a model which has been recommended for the biomedical domain: *full-domain generalization* followed by *record suppression* [6, 7].

**Fig. 1:**
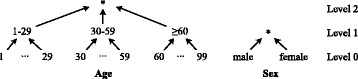
Examples for generalization hierarchies. The figure shows generalization hierarchies for two typical high-risk attributes: age and sex

Generalization is performed with user-defined hierarchies, which are transformation rules that reduce the precision of attribute values in a step-wise manner. As can be seen in Fig. 1, each hierarchy consists of a set of increasing levels, which specify values with increasing coverage of an attribute’s domain. Full-domain generalization means that the values of an attribute in all records are generalized to the same level of the associated hierarchy.

Generalization makes the records of a dataset less distinguishable, which reduces privacy risks. However, there may be records which cannot easily be generalized in such a way that they become indistinguishable from other records. To prevent that a large amount of generalization must be applied to the overall dataset, such outliers can be suppressed. This increases the quality of output data significantly [8]. Typically, the fraction of records that can be suppressed by a de-identification algorithm is restricted by specifying a so-called *suppression limit*.

### Solution space

Based on full-domain generalization, the solution space of potentially privacy-preserving transformations for a dataset is given by the set of all possible combinations of generalization levels for each attribute. Each such combination is also called a *de-identification policy*. The structure containing all policies for a given dataset is called a *generalization lattice*.

**Fig. 2:**
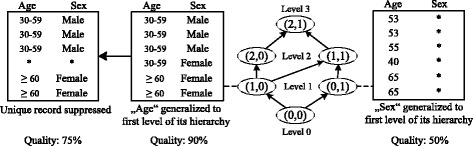
Example showing de-identification policies represented as a generalization lattice. The figure shows a generalization lattice constructed with the example hierarchies. It also shows the results of two de-identification policies and the resulting data quality

Figure 2 shows an example, which relates the concept of de-identification policies to an example dataset with attributes sex and age. The original dataset is at the bottom (0,0), whereas the policy specifying maximal generalization (2,1) is at the top. The figure further shows the output of applying the policies (1,0) and (0,1).

### Privacy models

The first objective in data de-identification is to minimize privacy risks. Typically, it is assumed that the attacker tries to link the disclosed dataset with some form of background knowledge. The attributes which can be used for linkage are called *quasi-identifiers*. Without loss of generality, we will in the remainder of this article focus on quasi-identifiers only.

Re-identification risks are typically modeled as some form of measure for the accuracy with which records from the background knowledge can be linked to records in the dataset. The most prominent model is *k*-anonymity, which enforces an upper bound of $\frac {1}{k}$ on the re-identification risk of all records [9–11]. A dataset is *k*-anonymous if each of its record cannot be distinguished from at least *k*−1 other records. Each set of indistinguishable records is called an *equivalence class*.

An example is shown in Fig. 2. The result of applying the de-identification policy (1, 0) to the original dataset and then suppressing the unique record is shown on the left side. The dataset fulfills the 2-anonymity model, which guarantees a maximal re-identification risk of 50 % for each record.

### Quality models

The second objective in data de-identification is to maximize data quality (or utility), which means that it must be measured. In the remainder of this article, we will use the model by Iyengar, which measures the extent to which the domains of the attributes are covered by a dataset [12]. For categorical variables, the basic idea is to use the generalization hierarchy for each attribute to determine the relative number of values from an attribute’s domain which is covered by the given (potentially transformed) value. These fractions are then summarized for all values in the dataset and normalized. The model is defined for continuous attributes as well. In this case, it is required that each node in the hierarchy defines an interval over the attribute’s domain. A value which has been suppressed covers the complete domain of the attribute, analogously to full generalization, and the fraction thus becomes one. For ungeneralized values the fraction is zero. The measure returns values in the range [0,1], where the original dataset has a quality of 100 %, while a transformed dataset in which all attribute values have been removed (either by generalization or suppression) has a quality of 0 %. In the examples shown in Fig. 2, quality according to the model by Iyengar is also indicated (75 %, 90 % and 50 % from left to right).

### Search strategies

Both objectives, i.e. minimizing re-identification risks while maximizing data quality, are conflicting. When using data de-identification algorithms this contradiction is typically resolved by letting a human decision maker define a preference on one of the optimization goals by specifying disclosure risk thresholds. An example is a parameterization for the *k*-anonymity model. What remains is a simpler optimization problem in which the objective is to make sure that risk thresholds are met while data quality is maximized. This is also called a priori disclosure risk control. With full-domain generalization this is implemented by searching the set of all de-identification policies, i.e. the generalization lattice, to find a solution which meets the privacy requirements and which is of high quality. As checking a de-identification policy for resulting privacy risks and data quality is an expensive operation, parts of the search space must be excluded to maintain scalability. This is typically implemented by using results obtained during the search process to *predict* properties of the output of de-identification policies in terms of privacy (first objective) or data quality (second objective). We can distinguish between three different types of algorithms.

**Type 1: Prediction using complete information**. These types of algorithms try to maximize the number of policies which can be excluded by using all information which has already been obtained during the search process. For this purpose, they maintain an in-memory graph structure which represents the generalization lattice. When a property can be predicted to other policies based on information obtained about the currently evaluated policy, the graph is traversed and the property is assigned to the according policies (this process is called *predictive tagging*). Typical examples are globally-optimal algorithms, such as Optimal Lattice Anonymization (OLA) [6] and Flash [13].

**Type 2: Prediction using partial information**. Algorithms from this category use prediction to exclude parts of the search space. However, the knowledge about properties of policies which have not yet been processed is only maintained temporarily. For example, properties of an evaluated policy are used while processing a related subset of the solution space. No explicit representation of the generalization lattice is required. Typically, such algorithms do not classify the complete search space and they cannot guarantee to find an optimal solution. The Lattice-Based Search (LBS) algorithm proposed in [14] is a good example.

**Type 3: No prediction**. Algorithms of this type are usually heuristics which have been proposed for processing high-dimensional or high-volume data. Examples are DataFly [15] and IGreedy [16]. They do not maintain a representation of the solution space. Large parts of this space are excluded from the search process. However, exclusion is not based on prediction and they cannot make any guarantees about the quality of the solution returned.

## Objective

In recent years, we have put extensive efforts into developing ARX, an open source de-identification tool for biomedical data [17]. As a search strategy, we have implemented an algorithm which uses prediction based on complete information [13], because with such methods it is possible to provide users with guarantees about the quality of output data. ARX is built around a highly scalable runtime environment which can handle very large datasets [8]. However, when using the tool in real-world setups, for example with longitudinal data from research registries [18], we quickly ran into scalability issues. Typically, the number of attributes in biomedical datasets which need to be protected is rather high. For example, the HIPAA Privacy Rule lists 18 different such attributes. Scalability becomes a problem because the size of generalization lattices is exponential in the number of attributes which need to be generalized (see Section “Results”). In addition to impractical memory requirements, the worst-case time complexity of predictively applying a property to de-identification policies is also proportional to the size of the lattice. Therefore this process often induced a significant overhead, even for data with only a few attributes.

The aim of the work presented in this article, was to push lattice-based data de-identification algorithms to their limits. The basic idea is to employ a highly optimized representation of the solution space. Our approach combines the mathematical concept of antichains with a data structure inspired by prefix trees to represent properties of a large number of de-identification policies while requiring only a minimal amount of information to be stored. To analyze the improvements which can be achieved by adopting our method, we have integrated it into ARX, where it serves as the basis of an existing algorithm of type 1. We have also implemented a simple search algorithm, which combines the ability to handle large search spaces typically provided by algorithms of type 2 and type 3 with the pruning capabilities of type 1 algorithms. This is a first step towards methods which fully exploit our approach.

## Methods

### Preliminaries and formalism

We denote the attributes $\mathcal {A}$ of the dataset that is to be de-identified as an *m*-tuple (*a*_1_,*a*_2_,…,*a*_*m*_), where *m* is the number of attributes. We denote the generalization lattice with $\mathcal {G}$ and call every $x \in \mathcal {G}$ a *de-identification policy* or *transformation*. Matching the *m*-tuple specifying the dataset’s attributes, each policy $x \in \mathcal {G}$ is an *m*-tuple of numbers (*x*_1_,*x*_2_,…,*x*_*m*_), where each number *x*_*i*_ represents a specific generalization level for the attribute *a*_*i*_, 1≤*i*≤*m*. We denote the *height* of the hierarchy for an attribute *a*_*i*_ with *h*_*i*_, which means that it consists of the generalization levels 0 to *h*_*i*_−1. Any policy $(x_{1}, x_{2}, \ldots, x_{m}) \in \mathcal {G}$ specifies generalization levels that are within these limits, i.e., 0≤*x*_*i*_<*h*_*i*_ for all 1≤*i*≤*m*.

Together with the relation *x*≺*y* for $x,y \in \mathcal {G}$ the solution space $\mathcal {G}$ forms a *partially ordered set* (or *poset*). The relation *x*≺*y* is defined so that for *x*=(*x*_1_,*x*_2_,…,*x*_*m*_) and *y*=(*y*_1_,*y*_2_,…,*y*_*m*_), *x*≺*y* if and only if *x*_*i*_≤*y*_*i*_ for all 1≤*i*≤*m*. This means that *x* only defines generalization levels that are less than or equal to the levels defined by *y*. This poset is a *bounded lattice* in which *top* =(*h*_1_−1,*h*_2_−1,…,*h*_*m*_−1) is the greatest element and *bottom* =(0,0,…,0) is the least element [19]. If *x*≺*y* we call *x* a *specialization* of *y* and *y* a *generalization* of *x*. The *rank* of an element *x* is the sum of its components, i.e. $rank(x) = \sum _{1 \leq i \leq m}x_{i}$, and the structure thus forms a *ranked poset* [19]. In the example from Fig. 2 the search space is illustrated with a *Hasse diagram*, which is a directed graph where each node represents a single policy that is connected to all of its direct specializations and generalizations [19]. Each *level* of the graph contains a set of policies with equal rank.

While a policy $x \in \mathcal {G}$ defines a vector of generalization levels, this information can also be used to represent transformation models that go beyond full-domain generalization. In this work, we focus on full-domain generalization followed by record suppression where a transformation *x* is applied to the dataset in the following manner:

- **Step 1:** Generalize the dataset according to the generalization levels defined by *x*.
- **Step 2:** Suppress all entries in all equivalence classes that do not fulfill the given privacy model.
- **Step 3:** If the number of suppressed entries is within the given limit, the policy is a solution candidate.

As a consequence, a policy *x* uniquely identifies a specific combination of generalization and suppression that is applied to the input dataset.

### Predictive properties

The basic idea of *predictive properties* is that some properties of the output of data transformations in terms of privacy and quality can be predicted from the results obtained for other transformations. We will distinguish between two types of predictive properties: *1)* a property *P↑* of the output of a transformation $x \in \mathcal {G}$ is inherited to the outputs of all generalizations of *x*. More specifically, this means that if the output of applying the transformation *x* results in a dataset with property *P↑*, the outputs of all transformations $y \in \mathcal {G}$ with *x*≺*y* will also have property *P↑*. Analogously, *2)* a property *P↓* of the output of a transformation $x \in \mathcal {G}$ is inherited to the outputs of all specializations of *x*. This means that if the output of applying the transformation *x* results in a dataset with property *P↓*, the outputs of all transformations $y \in \mathcal {G}$ with *y*≺*x* will also have property *P↓*.

Predictive properties can be used to exclude parts of the solution space from the search process. For example, if it is determined that the output of a given policy *x* does not fulfill the privacy model and it is known that this property is predictive to specializations, all specializations of *x* can not be a solution to the given de-identification problem either. We note that this concept is not new. We have already presented an overview of algorithms which make use of them in Section “Search strategies”. They have also been investigated in the context of several privacy models, e.g. in [9, 20–22] and [23], as well as for data quality models, e.g. in [12] and [24]. However, our experiences with ARX showed that only very few predictive properties are relevant in real-world setups and that they have to be used carefully. The reason is that prediction is tightly coupled to the generalization lattice but additional methods of data transformation, in our case record suppression, are also performed to improve output quality [8].

#### Insufficient protection against re-identification

The distinguishability of records will always decrease monotonically when the amount of generalization is increased, even when additional record suppression is performed [9, 20]. This means that the property of meeting re-identification risk thresholds is predictive within the solution spaces investigated in this article. The consequence is that both the property of *fulfilling k-anonymity* and the property of *not fulfilling k-anonymity* can be predicted, to generalizations and specializations, respectively. We note however that in contrast to previous work, e.g. globally-optimal type 1 algorithms such as OLA [6] and Flash [13], the predictability of the property of fulfilling *k*-anonymity cannot be used to exclude transformations from the search process. The reason is that, within the transformation model investigated in this article, data quality is not monotonic, which will be explained in more detail in the following section.

#### Insufficient data quality

The basic idea of this predictive property is to make use of the fact that data quality decreases monotonically with increasing degrees of generalization. In a generalization lattice this means that if a policy $x \in \mathcal {G}$ results in a dataset with quality *u*, all generalizations of *x* will result in datasets with quality less than or equal to *u* [6, 8]. However, within the transformation model investigated in this article, this is not the case. The reason is that increasing the amount of generalization may reduce the required amount of suppression, which may lead to an increase in data quality [8].

However, an upper bound for the quality of the output of a policy *x* can be derived by realizing that data is transformed in a two-step process. First, it is generalized according to the levels specified by *x*, resulting in a dataset with quality *u*^′^. Next some records may be suppressed, resulting in a dataset with quality *u*≤*u*^′^. Hence, *u*^′^ is an upper bound for *u*. The value of *u*^′^ solely depends on the application of generalization and all common models for data quality decrease monotonically with increasing full-domain generalization [8].

By adopting a method originally proposed by Bayardo et al. [24], the fact that these upper bounds are monotonic can be used to construct a property that is predictive to generalizations. During the search process, the upper bounds of the qualities of all policies which are processed are compared with the quality of the best output that is currently known. If the value of the bound *u*^′^ for a policy *x* is already lower than the quality of the current optimum, which is the result of applying generalization *and* suppression, all generalizations of *x* can be excluded.

As an example, we consider the datasets from Fig. 2 and assume that the dataset at the outer left side of the figure is the result of the best de-identification policy discovered so far. As we have illustrated previously, it has a data quality of 75 %. When we evaluate the policy (0,1) we realize that when only using generalization it already results in a dataset with a quality of only 50 %: one attribute remains unchanged (*age*) while one attribute is completely removed from the dataset (*sex*). As a consequence, the policies (1,1) and (2,1) can be excluded from the search process. They all specify that the attribute *sex* needs to be completely generalized and they will thus result in datasets with a quality of not more than 50 % as well.

### Efficient management of predictive properties

#### Basic idea

The size of a generalization lattice grows exponentially with the number of attributes which are to be generalized (see Section “Results”). To avoid the need to represent it, we suggest to maintain information about predictive properties only implicitly. We will describe our method for a property *P↑* that is predictive to generalizations. The concept analogously applies to properties that are inherited to specializations. The basic idea is to keep a list *L*(*P↑*) of all transformations for which a given predictive property *P↑* has been discovered. When determining whether a transformation $x \in \mathcal {G}$ is associated with the property we compare it with all transformations in the list and check whether any of the stored transformations *y*∈*L*(*P↑*) is a specialization of *x*, i.e., *y*≺*x*. If this is the case, the transformation is associated with the property as well.

#### Antichain invariant

Two elements $x \in \mathcal {G}$ and $y \in \mathcal {G}$ are *comparable* if *x*≺*y* or *y*≺*x*. An *antichain* is a subset of the elements of any $X \subseteq \mathcal {G}$ which are pairwise incomparable [19]. Any list of transformations *L*(*P↑*) for a property *P↑* specifies that this property is associated to a certain subset of the transformations in the search space. It is easy to see, that it is not necessary to have two transformations in the list which are comparable, as one of these two transformations will associate the property to the other transformation (and thus also to its generalizations) anyway. To enforce this invariant, we simply do not add a transformation to the list *L*(*P↑*) if it is already associated with *P↑*. Moreover, when an element *x* is added to the list we remove all of its generalizations, i.e., all *y*∈*L*(*P↑*) with *x*≺*y*. As a consequence, our approach guarantees that any list of transformations forms an antichain.

#### Implementation

Our implementation uses prefix trees to maintain an antichain of a given set of elements. In contrast to scanning a simple list and comparing a transformation with all of its entries, this structure enables us to exclude elements from this search process. The root node of a tree represents the predictive property, which is linked to a set of nodes that define generalization levels for the first attribute in the dataset *a*_1_. Their children represent generalization levels for the second attribute *a*_2_ and so on. Each path from the root node to a leaf node represents exactly one transformation $x \in \mathcal {G}$. Our data structure supports *inserts* and *queries*. Each node *n* has several attributes:

1. *n*.*level*: Represents a specific generalization level for the attribute.
2. *n*.*children*: A set of child nodes, which define generalization levels for the next attribute.
3. *n*.*min*: The minimum of the total generalization levels (ranks) of all transformations defined by its direct or indirect children.

Analogously to the previous section, we will use a property that is predictive to generalizations to explain our approach. All concepts apply to properties that are inherited to specializations as well, by replacing the *"* ≤*"* operator with *"* ≥*"*, the *"* <*"* operator with *"* >*"* and *"min"* with *"max"*. We will first describe the querying process and then explain how data is inserted.

**Queries:** When querying a tree for an element *x*=(*x*_1_,…*x*_*m*_) we perform a *range query* by following all children *c*(*n,x,i*)={*m*∈*n*.*children* | *m*.*level*≤*x*_*i*+1_∧*m*.*min*<*rank*(*x*)} of a node *n* at dimension *i*. This means that, because we only need to consider specializations of *x*, we follow all children that define generalization levels that are less than or equal to the ones of the element *x*. When we reach a leaf node, i.e. *n.children*={}, we have found a predecessor of the element *x* and conclude that *x* is associated with the property. Note that we follow only nodes that represent transformations with a rank lower than *rank*(*x*), as only such elements can be specializations of *x*. As a consequence, we cannot find the element *x* itself. However, in our context this is not a problem, because we will never query for a transformation that we have inserted into the structure. If this type of operation needs to be supported, an additional *exact query* for *x* needs to be performed if the range query did not return a positive result. This can be implemented by traversing one path of nodes *m* with *m*.*level*=*x*_*i*_ at dimension *i*.

**Fig. 3:**
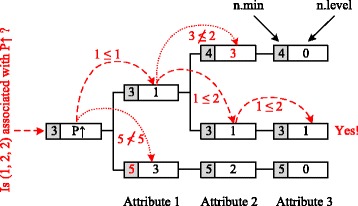
Example prefix tree for a property *P* *↑*. This figure displays an example of the data structure proposed in our work. It shows a tree after three elements have been inserted and it illustrates a querying process for the element (1,2,2)

As an example, we associate a predictive property *P↑* to the elements (1,1,1), (1,3,0) and (3,2,0). The resulting tree is shown in Fig. 3. We note that the three elements form an antichain, i.e. they are pairwise incomparable.

When checking whether *x*=(1,2,2) is associated with property *P↑*, we will first enumerate all children of the root node. We can exclude the second child, because *n*.*min*=5≥*rank*(*x*)=5. Next, we will enumerate all children of the element for the first attribute. Here, we can exclude the first child, because *n*.*level*=3>2=*x*_2_. When further traversing the path, we reach a leaf node and thus conclude that *x* is indeed associated with *P↑*.

**Inserts:** The basic insert operation is not different from insertions into prefix trees. However, for each node we maintain *n*.*min*, which is the minimum of the total generalization levels of all of its child nodes, and we make sure that our invariant holds, i.e. that the tree does only contain an antichain.

To enforce the invariant, as described previously, we first need to make sure that we only insert elements that are not already associated with the attribute. This can be implemented by querying the tree for an element before inserting it. However, in our context this is not necessary, because being associated with a predictive property means that a transformation is excluded from the search process and thus it will never be inserted. Secondly, after inserting an element *x* we need to remove all elements *y* with *x*≺*y*. This is implemented by performing a range query for *x* using the *"* ≥*"* operator (instead of *"* ≤*"*) before inserting the element. When reaching a leaf node, we remove it. While tracking back, we remove all inner nodes that do not have any child nodes anymore. We note that there is no need to update *n*.*min* for any node *n* that remains, because any child element *m* of *n* that is removed can only have *m*.*min*≥*n*.*min*.

### Effective pruning in large search spaces

Globally-optimal algorithms using complete information about predictive properties (type 1) typically implement in-memory materialization of the lattice. Their scalability can be improved by using our method to represent information about predictive properties only implicitly. However, they will still run into scalability issues when used with very large search spaces, as they usually iterate over all policies in the lattice [6, 13]. State-of-the-art heuristic search algorithms do not have this problem. However, they only use partial information (type 2) about predictive properties [14] or no predictive properties (type 3) at all [15, 16].

**Fig. 4:**
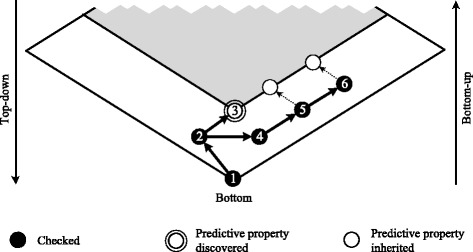
Example search process in a large solution space. The figure shows a generalization lattice, parts of which have been associated with a predictive property. It further describes a simple bottom-up search process, that leverages this information. Arrows and numbers denote the order with which the search algorithm traverses the solution space

As is indicated in Fig. 4, this means that algorithms for very large search spaces do not fully exploit the potential to narrow down the search space with predictive properties. In the figure, we consider a de-identification algorithm which traverses the solution space with a bottom-up best-first search. When a policy resulting in insufficient data quality is discovered (step 3), the algorithm can prune all generalizations from the search process. However, in subsequent steps (5 and 6) it may again reach the same part of the search space in which all output data will have insufficient quality. At this point, if the property is not predicted, some policies must be evaluated again.

To show that our approach enables us to use complete information about predictive properties even in very large search spaces, we have developed a very simple method, closely resembling the algorithm from the example. The basic idea is to perform a best-first branch and bound (BFS) bottom-up search trough the lattice. When a policy is evaluated, all generalizations which have not already been processed are evaluated and added to a priority queue. In the queue, all elements are ordered by data quality, from highest to lowest. At each step, the head is removed from the queue and evaluated. Policies resulting in data with insufficient quality are excluded from the search process. Complete information about all policies with this predictive property is managed using the method proposed in this article. We note that this algorithm combines characteristics of type 1 algorithms with the ability to handle very large search spaces as provided by algorithms of type 2 and 3.

### Experimental setup

In this section, we describe the setup of the experimental evaluation of our approach, which is based on an open source benchmarking environment [25]. We focused on measures against re-identification, because it is widely accepted that these are important in practice [26]. We used the *k*-anonymity privacy model with *k*=5, which is a typical parameter in the biomedical domain [27]. We used a suppression limit of 5 %, which is also common [6]. For measuring data quality, we used the model by Iyengar [12]. All experiments were performed on a desktop machine with a quad-core 3.1 GHz Intel Core i5 CPU and 8 GB of RAM running a 64-bit Linux 3.2.0 kernel and a 64-bit Oracle JVM (1.7.0).

We used six different real-world datasets, many of which have already been utilized for evaluating previous work on data de-identification. Low-dimensional datasets included *1)* an excerpt of 30,162 records (9 attributes) from the 1994 US census database (Adult), *2)* a dataset with 63,441 records (8 attributes) from the 1998 KDD competition (Cup), *3)* 100,937 records (8 attributes) about fatal traffic accidents from the NHTSA Fatality Analysis Reporting System (Fars), *4)* 539,253 records (9 attributes) from the American Time Use Survey (Atus), and, *5)* 1,193,504 records (9 attributes) from the Integrated Health Interview Series (Ihis). The sizes of the corresponding generalization lattices ranged from 12,960 policies for the Adult dataset to 45,000 policies for the Cup dataset. For experiments with high-dimensional data, we used 68,725 records from the American Community Survey (SS13ACS) [28]. Each record consisted of 15 attributes which are typically associated with a high risk of re-identification, such as demographics (e.g. age, marital status, sex), information about insurance coverage, social parameters (e.g. education) and health parameters (e.g. weight, health problems). With 15 attributes, the search space consisted of more than 113 million transformations.

## Results

### Complexity analysis

For a dataset in which *m* attributes are to be generalized with hierarchies of heights *h*_1_,…,*h*_*m*_, the solution space consists of $s = \prod _{i=1}^{m} h_{i}$ elements, which means that the number of transformations is exponential (with linear exponent) in the number of attributes ($2^{\mathcal {O}(m)}$). As a consequence, a large amount of memory is required to represent the search space and constructing the data structure in main memory and predictively applying properties to de-identification policies may induce a noticeable computational overhead.

Compared to using an in-memory graph structure, our implementation shifts the complexity of maintaining information about predictive properties from being dependent on the size of the search space *s* to being dependent on the number of transformations for which a property has been encountered. At any point during the search process, this number cannot be larger than the total number of transformations *c* that have been checked so far. In a theoretical worst-case, *c* may be equal to *s*, because all transformations may have been checked and found to have a predictive property. However, for large search spaces *c* can only be a tiny fraction of *s*.

When the generalization lattice is explicitly materialized, assigning a predictive property to a transformation (and its generalizations or specializations) is of worst-case complexity $\mathcal {O}(s)$. The reason is that the property may be inherited to all transformations in the space. On the other hand, determining whether a given transformation is associated with a predictive property is an $\mathcal {O}(1)$ operation, as it is simply a lookup into the structure.

Next, we analyze the complexity of maintaining a list of all transformations that have a certain property. Here, the worst-case complexity of storing a predictive property is $\mathcal {O}(1)$, as the transformation is simply added to the list. At a specific point during the search process, the worst-case complexity of checking whether a given transformation is associated with a property is $\mathcal {O}(c)$. We have thus replaced a data structure optimized for read-access with a data structure optimized for write-access. Moreover, we have reduced the worst-case complexity of the most complex operation of the structure from an impractical $\mathcal {O}(s)$ to $\mathcal {O}(c)$.

Finally, we consider the invariant enforced on the structure. Making sure that the list of transformations always contains an antichain, requires an additional scan of the list on every insert operation. As a consequence, the worst-case complexity of inserting an element increases from $\mathcal {O}(1)$ to $\mathcal {O}(c)$. While the general idea of the invariant is to reduce the complexity of the querying operation (as it reduces the number of elements stored in the structure), it is ineffective in the worst case, which is given if all elements that have been inserted already form an antichain.

The implementation proposed in this article does not use a list but a prefix tree. Moreover, the tree is further optimized by maintaining information about the stored elements’ ranks. As a consequence, potentially large parts of the elements stored in the structure can be ignored during inserts or queries. However, in terms of worst-case complexity this approach does not provide any benefits, as the tree may degenerate to a list.

### Experimental analysis

#### Evaluation of implementation options

In this section, we compare different options for implementing our approach to clearly show that each design decision presented previously improves performance in real-world settings. Table 1 shows a comparison of execution times obtained by de-identifying the different datasets with the globally-optimal Flash algorithm [13]. The reported performance numbers only include the time required to manage information about predictive properties (inserts and queries). *Option 1* is a simple list, *option 2* is a list that implements the invariant, *option 3* is the described tree structure, but without the optimization of storing minimal generalization levels, and, *option 4* is the method described in this article.

**Table 1:** Comparison of different implementations and optimizations. The table shows actual times spent with updating and querying the generalization lattice with different implementations as well as improvements in performance compared to closest less sophisticated implementation option

	Option 1	Option 2	Option 3	Option 4
Dataset	Simple list	List with antichain	Prefix tree with antichain	Optimized tree with antichain
Adult	0.247s	0.230s (6.88 %)	0.061s (73.48 %)	0.055s (9.84 %)
Cup	0.658s	0.525s (20.21 %)	0.232s (55.81 %)	0.218s (6.03 %)
Fars	0.364s	0.240s (34.07 %)	0.074s (69.17 %)	0.071s (4.05 %)
Atus	0.300s	0.185s (38.33 %)	0.095s (48.65 %)	0.081s (14.74 %)
IHIS	0.437s	0.251s (42.56 %)	0.124s (50.60 %)	0.093s (25.00 %)
SS13ACS	21.730s	15.032s (30.82 %)	1.861s (87.62 %)	1.797s (3.44 %)

The SACS13 dataset contained 10 attributes.

It can be seen that each more sophisticated implementation significantly reduced the time required for maintaining information about the solution space. The largest improvement was achieved by replacing the list with a prefix tree. In total, execution times were reduced by up to more than one order of magnitude (SS13ACS dataset).

#### Evaluation with a globally-optimal algorithm

In this section, we analyze how globally-optimal algorithms which use complete information about predictive properties can benefit from adopting our approach. In the experiments we compared our existing implementation of the Flash algorithm, which uses an *explicit* in-memory graph structure representing the generalization lattice, with a revised implementation which uses the *implicit* approach proposed in this article.

Using the five low-dimensional datasets, the implicit implementation consistently outperformed the explicit implementation. We measures speed-ups between 5 % and 23 % combined with a reduction of memory required to represent the search space between 7 % and 50 %. The improvement in memory consumption roughly corresponded with the improvement in execution times.

Table 2 summarizes statistics collected about predictive properties during the execution of the experiments. It can be seen that, in each experiment, less than 10 % of the policies in the solution space needed to be evaluated by the algorithm to find the optimal solution. This was achieved by utilizing the two predictive properties described in Section “Predictive properties”. As can be seen, most output datasets were found to have insufficient quality. For this property the hit rate, which is the relative number of policies for which a query to the prefix tree returned a positive result, was also relatively high. In contrast, only few policies provided insufficient protection against re-identification. Here, the sizes of the antichains were also significantly higher than for the other property.

**Table 2:** Statistics about predictive properties obtained for low-dimensional datasets

Data	Trans.	Checked	Property		Inserts	Hits	Antichain
Adult	12,960	1,180 (9.10 %)	Insufficient quality	*↑*	1,062	74.69 %	73.54 %
			Insufficient protection	*↓*	887	15.37 %	93.01 %
Cup	45,000	1,524 (3.39 %)	Insufficient quality	*↑*	1,435	80.93 %	76.31 %
			Insufficient protection	*↓*	1,172	24.75 %	96.84 %
Fars	20,736	1,342 (6.47 %)	Insufficient quality	*↑*	1,161	75.44 %	61.84 %
			Insufficient protection	*↓*	752	10.81 %	88.43 %
Atus	34,992	1,022 (2.92 %)	Insufficient quality	*↑*	903	82.53 %	59.25 %
			Insufficient protection	*↓*	561	5.23 %	95.37 %
Ihis	25,920	1,574 (6.07 %)	Insufficient quality	*↑*	1,341	73.58 %	42.80 %
			Insufficient protection	*↓*	679	7.91 %	90.28 %

We report the size of the solution space, the percentage of transformations checked as well as the number of inserts, the number of hits and the maximal size of the antichain for each predictive property. The size of the antichain is expressed relatively to the number of inserts

**Fig. 5:**
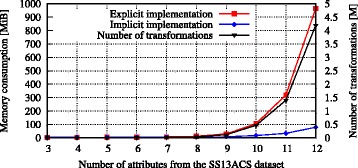
Memory consumption when de-identifying high-dimensional data. The figure shows the memory consumed by an explicit and by an implicit representation of the solution space when de-identifying the SSACS13 dataset

We used the high-dimensional datasets to further analyze effects on memory consumption. Figure 5 shows the memory required when using the explicit and the implicit representation of the generalization lattice. As can be seen, the memory consumption of the explicit implementation correlated with the number of de-identification policies in the search space. In contrast, the implicit representation consumed significantly less memory, by almost one order of magnitude (e.g. ∼100 MiB instead of ∼1 GiB with 12 attributes). When using the explicit implementation, the Flash algorithm was not able to process datasets with more than 12 attributes, as it ran out of memory.

#### Evaluation of the BFS algorithm

In this section, we analyze the performance of our simple best-first branch and bound bottom-up search algorithm presented in Section “Effective pruning in large search spaces”. We start by evaluating its suitability as a globally-optimal algorithm.

**Fig. 6:**
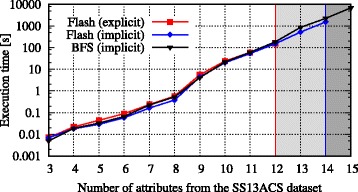
Execution times when de-identifying high-dimensional data. The figure shows the performance achieved with the Flash algorithm using an explicit and an implicit representation of the solution space. Moreover, it shows numbers obtained with the BFS algorithm using an implicit representation of the solution space

Figure 6 shows a comparison of BFS with the two variants described of Flash when processing the SS13ACS dataset with increasing dimensionality. It can be seen that the performance of all three approaches was roughly equivalent. However, with an explicit representation of the solution space, Flash was not able to handle more than 12 attributes. With an implicit implementation the Flash algorithm was able to handle up to 14 attributes. The BFS algorithm could handle up to 15 attributes for which it returned an optimal solution after about 6850 seconds.

To show that the BFS method benefits from its use of predictive properties, we performed the same experiments without using prediction. The results are shown in Table 3. As can be seen, using predictive properties improved the execution times of the BFS strategy in every experiment. The strength of the effect varied, as using different attributes also resulted in very different search problems. In general, the complexity of a de-identification problem increases when increasing the number of attributes. This effect is also reflected by our results. The performance improvements varied between 3 *%* and almost 20 *%*. We note that larger improvements have been achieved for data of higher dimensionality, where execution times are generally longer and performance optimization is thus more important.

**Table 3:** Impact of predictive properties on execution times when de-identifying high-dimensional data. The figure shows the performance achieved with the BFS algorithm when using or not using predictive properties

Attributes	4	5	6	7	8	9
Without prediction [s]	0.021	0.035	0.071	0.229	0.545	4.47
With prediction [s]	0.019	0.033	0.067	0.223	0.533	4.13
Improvement	9.52 %	5.71 %	5.63 %	2.62 %	2.20 %	7.68 %
Attributes	10	11	12	13	14	15
Without prediction [s]	24.02	70.19	215.55	1032.19	2811.32	8259.83
With prediction [s]	21.69	61.24	185.51	858.41	2248.37	6843.33
Improvement	9.72 %	12.75 %	13.93 %	16.83 %	20.02 %	17.15 %

Table 4 summarizes statistics collected about predictive properties during the execution of the BFS algorithm. It can be seen that the percentage of policies from the solution space which were evaluated by the method decreased when the number of attributes was increased. Although the algorithm made use of only one predictive property, its pruning strategy was highly effective. The hit rate increased and the size of the antichain decreased with increasing dimensionality. The numbers show that our approach works well, even for high-dimensional data. With 15 attributes, our simple BFS search strategy was able to determine the optimal solution out of 113 million transformations.

**Table 4:** Statistics about predictive properties obtained for the high-dimensional datasets

Attributes	Transformations	Checked	Inserts	Hits	Antichain
3	96	12 (12.50 %)	4	17.39 %	75.00 %
4	480	50 (10.42 %)	18	20.87 %	55.56 %
5	1,440	89 (6.18 %)	34	22.84 %	44.12 %
6	4,320	177 (4.10 %)	61	25.13 %	36.07 %
7	12,960	449 (3.46 %)	157	28.06 %	31.85 %
8	38,880	820 (2.11 %)	284	29.99 %	24.65 %
9	116,640	3,872 (3.32 %)	1,187	34.26 %	22.16 %
10	466,560	15,858 (3.40 %)	4,486	36.80 %	22.78 %
11	1,399,680	32,507 (2.32 %)	10,119	37.70 %	20.43 %
12	4,199,040	76,679 (1.83 %)	25,211	38.36 %	18.84 %
13	12,597,120	265,762 (2.11 %)	85,303	38.74 %	19.59 %
14	37,791,360	626,383 (1.66 %)	199,747	39.15 %	20.75 %
15	113,374,080	1,634,751 (1.44 %)	514,863	39.31 %	20.17 %

We report the size of the solution space, the percentage of transformations checked as well as the number of inserts, the number of hits and the maximal size of the antichain for the predictive property *insufficient quality*. The size of the antichain is expressed relatively to the number of inserts

We emphasize that all algorithms studied in the previous paragraphs and sections are globally-optimal algorithms of type 1, and that all of them returned the same results. The results of the BFS algorithm demonstrate that our approach allows implementing methods which are able to exclude parts of very large search spaces using predictive properties and complete information. This means that the BFS method combines properties of type 2 and type 3 algorithms with properties of type 1 algorithms.

**Fig. 7:**
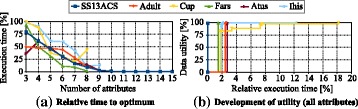
Converting the BFS algorithm to a heuristic de-identification method. Subfigure **a** shows that the relative time required to find the optimal transformation decreases significantly when the number of attributes is increased. Subfigure **b** shows that the quality of the solution increases very fast during the search process. Both properties of the BFS algorithm indicate that it delivers good results quickly, which makes it well suited as a heuristic strategy that terminates after a user-defined amount of time

In the remainder of this section, we will analyze the suitability of the BFS method as a heuristic search strategy. Figure 7a shows the time required by the method to discover the global optimum relative to its total execution time for all six datasets. It can be seen that, as a general trend, the fraction of time required to find the optimum dropped significantly when the number of attributes in the datasets was increased. For example, the optimal solution for the SS13ACS dataset with 15 attributes was found within the first 500 ms, which is only about 0.007 % of the time required to search the complete solution space (almost two hours, see above). In all cases, the BFS method found the optimal transformation in less than 25 seconds. This is also reflected by the development of the quality of the solution over time, which is shown in Fig. 7b. Here, each dataset contained all available attributes.

Table 5 shows a comparison of the BFS method with the Improved Greedy Heuristic (IGreedy), which is a state-of-the-art heuristic de-identification algorithm [16]. We converted BFS into a heuristic by simply terminating it after the amount of time required by IGreedy. The table shows the improvement in data quality provided by our method. Analogously to the experiments shown in Fig. 7, we have performed 45 experiments using all datasets and varying subsets of their attributes. In 9 experiments, BFS and IGreedy returned the same result. In 34 experiments, our approach returned a result with improved quality, between 1.36 % and 4.58 % on average, and by more than 12 % in one experiment. In 2 experiments (Cup dataset with 8 and 9 attributes), our approach did not find a solution within the time required by IGreedy. This result corresponds with the rather long time required to find the optimum for Cup in Fig. 7. However, in both cases executing our method for only 14 seconds would have returned an output dataset with higher quality than the result of IGreedy.

**Table 5:** Improvement in quality provided by data returned from the BFS algorithm compared to results of the IGreedy method. The BFS algorithm has been terminated after the time required by IGreedy. In two experiments with Cup, the BFS method did not return a result

	Adult	Cup	Fars	Atus	Ihis	SS13ACS
Minimal improvement	0.00 %	0.04 %	0.00 %	0.79 %	0.00 %	0.00 %
Maximal improvement	7.69 %	2.38 %	6.70 %	5.46 %	12.44 %	6.04 %
Average improvement	1.92 %	1.42 %	1.36 %	1.99 %	4.58 %	1.74 %

## Discussion

### Principal results

In this article, we have presented an efficient method for representing predictive properties of a large number of de-identification policies while requiring only a minimal amount of information to be stored. We have also developed a simple best-first branch and bound search algorithm which is a first step towards methods which fully exploit our approach by using complete information about predictive properties in very large search spaces.

When integrated into an existing de-identification algorithm, our approach reduced memory requirements by up to one order of magnitude and execution times by up to 25 %. This allowed us to increase the size of solution spaces which could be processed by almost a factor of 10. When using the BFS method as a globally-optimal algorithm, we were able to further increase the size of the solution space by a factor of three. When using BFS as a heuristic strategy, it outperformed a state-of-the-art algorithm by up to 12 % in terms of the quality of output data.

We emphasize that full-domain generalization is an important transformation model for de-identifying biomedical data, because it is *truthful* (i.e. non-pertubative) [29], produces datasets that are suited for being analyzed by epidemiologists [6] and it is interpretable and easy to understand by non-experts [11]. The scalability issues investigated in this article are of high practical relevance as the number of attributes in biomedical datasets which need to be protected is often high. All methods described in this article have been implemented into the open-source de-identification tool ARX [17].

### Comparison with prior work

In the previous sections, we have already put our approach into context with prior work on algorithms using full-domain generalization. In this section, we will compare the methods used in this article to other relevant work on models for measuring data quality and privacy, transformation methods and de-identification algorithms.

Our method for predicting that the output of de-identification policies only provides insufficient data quality requires monotonic lower bounds to be available for the data quality model. We emphasize that we have found such lower bounds for all common quality models, including KL-Divergence [30] and Non-Uniform Entropy [6], which have also been recommended for de-identifying health data [5, 11]. In the ARX tool, we have implemented this predictive property for eight different quality models, including all methods mentioned in this article [17].

Our method for predicting that the output of de-identification policies only provides insufficient privacy protection requires that risks decrease monotonically with increasing generalization. We note that this is the case for all metrics for re-identification risks which are typically used in practice [31]. In this work, we have used the *k*-anonymity model as a well-known example. In addition to privacy models focusing on re-identification, similar predictive properties can be found for other models as well. For example, *ℓ*-diversity implies *ℓ*-anonymity [30, 32] and the property can therefore also be used to optimize protection methods against attribute disclosure. In ARX, privacy requirements can be defined as arbitrary combinations of different privacy models, and any predictive property provided by at least one of the models will be exploited by the tool [17].

In this work we have focused on data de-identification with full-domain generalization. Other works have investigated algorithms using different transformation models. For example, Fung et al. [33] and Xia et al. [5] have developed approaches using subtree generalization, which is more flexible than full-domain generalization. However, the results are complicated to analyze [34]. Analogously to the approach presented in this article, Fung et al. focus on finding a single solution which maximizes data quality [33]. In contrast, Xia et al. aim to efficiently construct a risk-utility frontier [5], which is a set of policies that offer a good balance between privacy and data quality. We note that risk-utility frontiers are not related to the antichains used in this article. However, our approach can be used as a building block for balancing risks and quality, for example by repeatedly executing the algorithm with different risk thresholds to construct a risk-utility frontier [35]. Several algorithms have also been developed which transform data with microaggregation, where the values within an equivalence class are transformed using aggregate functions, such as the arithmetic mean. Examples include the approach by Domingo-Ferrer and Torra [36] and the approach by Soria et al. [34]. Microaggregation is not truthful, however. An additional line of research involves methods using local recoding with generalization, for example, the approach by Goldberger and Tassa for *k*-anonymity and *ℓ*-diversity [37]. With these methods, quality can be improved but results are also complicated to analyze [34]. The ARX tool supports full-domain generalization, record suppression, local recoding and microaggregation [17].

### Limitations

Our experiments showed that our method performs far better in real-world settings than the worst-case complexities from Section “Complexity analysis” suggest. However, analyzing average-case or amortized worst-case complexities of our method is difficult. The main reason is that the mathematical foundations of generalization lattices are not well understood. The closest structure that we could find in the literature is the so called *chain product poset* studied by Carroll et al. [38]. It is a special case of the structure considered here, with *h*_1_=*h*_2_=…=*h*_*m*_=*h*. Let *a* be the maximal size of an antichain of the chain product poset constructed from a generalization lattice $\mathcal {G}$ by choosing *h*=*max*(*h*_1_,*h*_2_,…*h*_*m*_). It follows that *a* is an upper bound for the maximal number of elements that may be contained in our data structure. In any chain product poset the middle level, which contains all elements with rank $\lfloor \frac {(h-1)}{2} \cdot m \rfloor $, is an antichain of maximal cardinality [38]. The paper cites an article by Mattner et al. which shows that the size of the middle level, which equals *a*, is $h^{m} \sqrt {\frac {6}{\pi (h^{2}-1)m}}(1+o(1))$ [39]. This is no improvement over $\mathcal {O}(c)$. Also, the average size of an antichain in such lattices is unknown. The most well studied lattice is the *Boolean lattice*, which is given by *h*=2. Even for this simple structure, the total number of antichains can only be estimated to date [38].

Also, from an implementation perspective, we could not find better bounds on the complexity of operations on prefix trees. There are some works on the average-case complexity of querying prefix trees, e.g. [40, 41], but their results are not applicable to our context, most importantly because we perform range queries and not look-ups. Although specialized prefix trees for range queries have been studied in the literature as well, e.g. [42, 43], results on their complexities are also not applicable to our work. The reason is that these data structures have been designed for managing totally ordered sets while the focus of our work lies on elements that are only partially ordered.

While the work presented in this article improves the scalability of de-identification algorithms for high-dimensional data, the method is not well suited for de-identifying data with a very high number of attributes (e.g. more than 50). The reason is that complex inter-attribute relationships will often result in unacceptable reduction of data quality [44]. One solution to this problem is to treat the data as transactional, i.e. set-valued, which is a way to remove inter-attribute relationships. Specific privacy models have been proposed for such data, for example *k*^*m*^-anonymity [45] and (*k, k*^*m*^)-anonymity [46]. In future work, we plan to integrate these methods into ARX as well.

## Conclusions

The work described in this article shows that implementing methods of data de-identification for real-world applications is a challenging task. We investigated a problem often faced by data custodians: a lack of scalability of de-identification software when used with datasets having realistic schemas and volumes. We have proposed a solution with which the scalability of existing implementations and algorithms can be improved and which enables the development of novel heuristic algorithms with improved pruning capabilities.

## Availability of data and materials

All datasets, generalization hierarchies and implementations of algorithms used in our evaluation are available online (https://github.com/arx-deidentifier/pruning-benchmark). All methods have also been implemented into the open source data anonymization tool ARX (http://arx.deidentifier.org). Moreover, the data structure for efficiently managing information about predictive properties is available as a separate open source software library (https://github.com/prasser/jhpl).
